# A Low-Power SAR ADC with Capacitor-Splitting Energy-Efficient Switching Scheme for Wearable Biosensor Applications

**DOI:** 10.3390/mi14122244

**Published:** 2023-12-15

**Authors:** Yunfeng Hu, Qingming Huang, Bin Tang, Chaoyi Chen, Lexing Hu, Enhao Yu, Bin Li, Zhaohui Wu

**Affiliations:** 1 Zhongshan Institute, University of Electronic Science and Technology of China, Zhongshan 528402, China; 2 School of Microelectronics, South China University of Technology, Guangzhou 510640, China

**Keywords:** SAR ADC, capacitor-splitting, common-mode shift, low complexity

## Abstract

A low-power SAR ADC with capacitor-splitting energy-efficient switching scheme is proposed for wearable biosensor applications. Based on capacitor-splitting, additional reference voltage V_cm_, and common-mode techniques, the proposed switching scheme achieves 93.76% less switching energy compared to the conventional scheme with common-mode voltage shift in one LSB. With the switching scheme, the proposed SAR ADC can lower the dependency on the accuracy of V_cm_ and the complexity of digital control logic and DAC driver circuits. Furthermore, the SAR ADC employs low-noise and low-power dynamic comparators utilizing multi-clock control, low sampling error sampling switches based on the bootstrap technique, and dynamic SAR logic. The simulation results demonstrate that the ADC achieves a 61.77 dB SNDR and a 78.06 dB SFDR and consumes 4.45 μW of power in a 180 nm process with a 1 V power supply, a full-swing input signal frequency of 93.33 kHz, and a sampling rate of 200 kS/s.

## 1. Introduction

**Figure 1:**
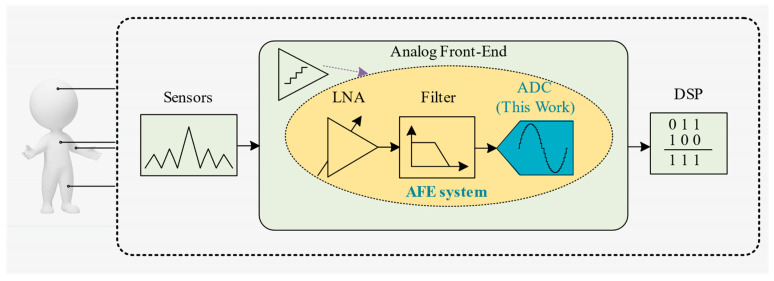
Block diagram of a wearable biosensor integrated circuit.

The rapid development of wireless sensors, portable electronic devices, and biomedical systems has led to ever-increasing performance needs for ADCs utilized in sensor interfaces, wearable medical devices, and other areas [1]. Among them, wearable biosensor applications have attracted more attention, with features such as lightweight, battery-powered or energy-harvesting circuit-powered, and low sampling frequency posing challenges for system design in terms of miniaturization and ultra-low power consumption [2]. Therefore, efforts have been made to develop low-power wearable biosensor applications or portable medical devices to extract and analyze signals [3,4] to efficiently reflect information such as human health status through early detection of biophysical signals. As shown in Figure 1, in a standard front-end readout system, single-ended biophysical potential signals such as nerve signals and electromyographic (EMG) signals are captured and pre-processed by sensors and AFE modules, and the ADC converts the analog signals that have been processed at the previous level into digital signals that can be processed by the CPU. ADCs play a key role in the system and consume a lot of power. Physiological signals are generally weak with frequency components lying between DC to few hundred Hertz and the amplitude varying from millivolt to microvolt. Thus, a moderate speed (sampling rate) converter is enough to handle these signals [5]. The frequency and amplitude ranges of common physiological signals such as electrocardiogram (ECG) signals, electromyography (EMG) signals, electroencephalogram (EEG) signals, and electrocorticography (ECoG) signals are shown in Table 1.

**Table 1:** Physiological signals and its frequency and amplitude ranges [6].

Type of Biological Signals	Frequency Range (Hz)	Amplitude (mV)
ECG	0.01 to 250	0.5 to 4
EMG	Up to 2 K	0.1 to 5
EEG	0.5 to 150	0.0005 to 0.3
ECoG	70 to 110	0.001 to 3

In recent years, the successive approximation register analog-to-digital converter (SAR ADC) has been vigorously developed in the context of the increasing demand for low-power applications due to their simple structure, smaller area, and lower power consumption [1,7,8,9,10,11,12,13,14,15]. The above advantages justify the use of SAR ADCs as converters in wearable biomedical devices that process physiological signals [6]. Therefore, the design of low-power SAR ADCs has attracted more and more attention, and the innovative designs have achieved higher performance and efficiency in circuits and systems, providing more efficient, high-performance, and high-reliability electronic devices for biosensor applications. In a SAR ADC, the main sources of energy consumption are the capacitive digital-to-analog converters (DACs), comparator, and SAR control logic [16]. The dynamic comparator does not consume static current, and the digital control logic receives benefits from technology scaling. Therefore, the capacitive DAC dominates the overall power consumption [17,18], especially when the resolution increases [19]. The switching energy can be greatly reduced by changing the structure of the capacitor array and the switching method of the capacitors [20].

Several low-power switching schemes have been aggressively introduced to make DACs consume less energy [10,11,12,13,14]. Compared to the conventional architecture [8], the monotonic switching scheme (set and down) based on capacitive top-plate sampling reduces the switching energy loss by 81.26% [9]. Compared to the conventional switching scheme, the V_cm_-based switching scheme [10], Wang et al. [11], HSRS [12], SMS [13], and Hu et al. [14] reduce the switching energy by 87.52%, 90.61%, 92.20%, 95.32%, and 95.34%, respectively. The monotonic downward switching scheme is proposed in [9], while V_cm_ is used in [10] for energy reduction. These DAC structures [10,11,12,13,14] consume substantially less energy, most of them require a high accuracy of V_cm_ to minimize the resultant error of DACs, or have a large common-mode shift [11,12,13,14]. Refs. [10,12,13] rely on three reference levels for each capacitor switch, so the complexity of the logic control of the DAC module is increased, and the difficulty of subsequent circuit implementation is increased, thereby limiting the overall power consumption reduction. Ref. [12] proposed a higher-side-reset-and-set (HSRS) switching scheme with an equally simple and energy efficient logic circuit but with limited energy savings.

To reduce the overall circuit complexity and power consumption of the SAR ADC, a capacitor-splitting low-complexity and energy-efficient DAC switching scheme with one LSB common-mode shift for SAR ADC has been designed. The capacitor-splitting structure is a method to reduce power and area. In this work, the capacitor-splitting structure, common-mode technique, and V_cm_ are combined and optimized to achieve the proposed switching scheme. Behavioral simulation of a 10-bit SAR ADC shows 93.76% less switching energy than the conventional method. The V_cm_-based tri-level switching scheme is more area-efficient because of the introduction of V_cm_ (equal to V_ref_/2). Ref. [13] achieved lower switching energy at the cost of high logic complexity. In the proposed switching scheme, only three reference levels are employed for the second least significant bit (Second LSB), while two reference voltages are assigned per capacitor for the remaining capacitors. This design choice significantly reduces the complexity of the capacitance array. Additionally, it is worth noting that the accuracy of V_cm_ has minimal impact on the accuracy of the digital-to-analog converter (DAC). It only affects the accuracy of the analog-to-digital converter (ADC) in the last two conversion cycles. The common-mode voltage of the capacitor array remains constant except for the LSB conversion, reducing the complexity of the comparator design. Thus, the proposed switching scheme is a good trade-off among switching energy, capacitor area, DAC output common-mode voltage, and ADC accuracy.

Due to the increasing demand for low-power biosensor electronics, many leading-edge switching schemes for DAC capacitor arrays are being applied to SAR ADCs in this field. Ref. [21] describes the design and calibration of an ultra-low power 14-bit 10 KS/s fully differential SAR ADC for biomedical applications. The integrated transient response of the ADC shows a power consumption of only 19.5 µW. Ref. [22] presents two low-power design techniques used for SAR ADC for the transmission of a physiological signal: a dual split switching, and a set and reset phase. The power consumption of the SAR ADC is 13.99 μW.

To validate the effectiveness and benefits of the proposed scheme, a 10-bit 200 kS/s SAR ADC circuit was simulated and analyzed using a 180 nm CMOS process with a supply voltage of 1 V. The behavioral simulation results revealed that the ADC achieved impressive metrics, including a signal-to-noise and distortion ratio (SNDR) of 61.77 dB, a spurious-free dynamic range (SFDR) of 78.06 dB, and a power consumption of 4.45 μW. These achieved metrics meet the requirements of most biosignal applications, showcasing the favorable energy efficiency of the proposed SAR ADC. The results clearly demonstrate the potential of the proposed DAC switching scheme in enhancing the performance of SAR ADCs. The innovation of this work lies in the effective combination of a capacitor-splitting structure and a third reference voltage, V_cm_, to achieve a high energy-efficient SAR ADC with a common-mode shift of one least significant bit (LSB) using a common-mode technique.

## 2. Design of the Proposed SAR ADC

**Figure 2:**
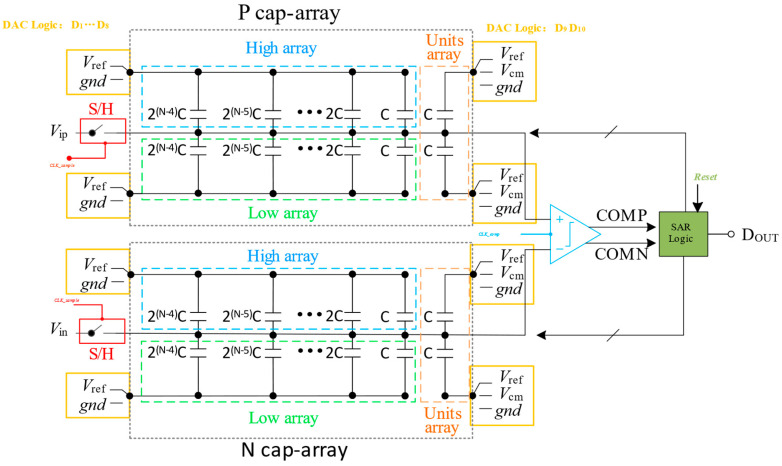
The proposed architecture of N-bit SAR ADC.

The structure of the N-bit SAR ADC is demonstrated in Figure 2. To reduce supply voltage noise and achieve effective common-mode noise rejection, we have implemented the fully differential architecture [23]. The key components of a SAR ADC include the sample-and-hold circuit, the comparator, the capacitive DAC, and successive approximation registers. The capacitive DAC is composed of a positive-phase capacitance array (P cap-array) and a negative-phase capacitance array (N cap-array). Each of these arrays is further divided into three sub-capacitor arrays: high array, low array, and units array. The high array and low array are binary weighted capacitor arrays. Based on the top-plate sampling technique, the analog input is sampled into the ADC through a sample-and-hold circuit. This process is controlled by the sampling switch clock, CLK_sample. Once the input is sampled, the SAR ADC employs a successive approximation logic algorithm to convert the analog input value into a digital output code. The comparator clock, CLK_comp, is driven by the reset signal. This clock controls the operation of the comparator, which compares the analog input voltage with the voltage generated by the DAC. The reset signal also triggers the SAR logic to initiate the conversion process, controlling the generation of individual bits. Detailed signaling control details are shown in Figure 2.

### 2.1. DAC Switching Scheme

**Figure 3:**
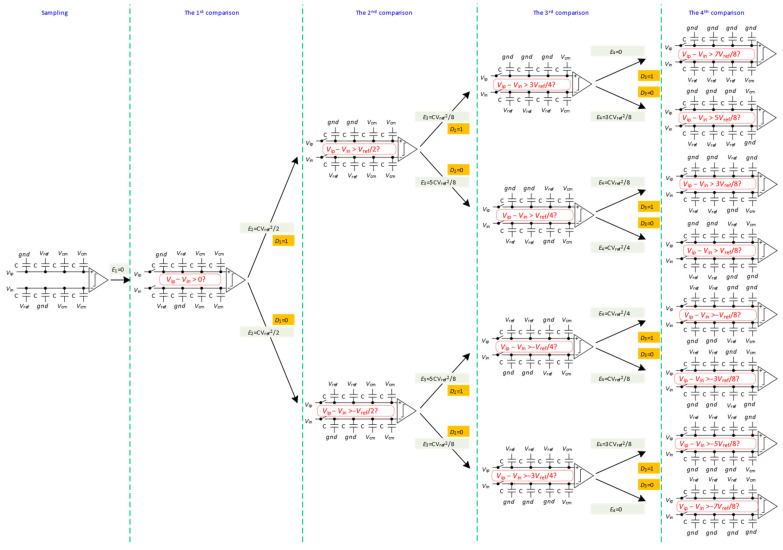
Switching procedure of 4-bit SAR DAC.

To improve energy efficiency and reduce power consumption, circuit complexity, and occupied area, a novel energy-efficient switching algorithm is proposed for a 4-bit SAR ADC, as illustrated in Figure 3. The gray area in the figure represents the switching of the reference voltage at each conversion stage. In the sampling state, top-plate sampling reduces DAC resolution requirements [9,24,25]. The operation of the ADC can be divided into five phases: sampling and 1st comparison, 2nd to (N − 2)th comparison, (N − 1)th comparison, and Nth comparison.

Sampling and 1st comparison: The input signal is sampled through the sampling switch on the top plates of all capacitors. The bottom plate of the capacitors in the high array is connected to the reference voltage (V_ref_), the bottom plate of the capacitors in the low array is connected to ground, and the bottom plate of the capacitors in the unit array is connected to the common-mode voltage (V_cm_). After sampling, the sampling switch is turned off, and the comparator performs the initial comparison without using any switching energy. The outcome of this first comparison is denoted as D_1_.

2nd to (N − 2)th comparison: According to the output of the previous comparison (D_i−1_), the corresponding capacitor in the high array on the high-voltage potential side will be switched from V_ref_ to ground. Similarly, the corresponding capacitor in the low array on the low-voltage potential side will be switched from ground to V_ref_. All other capacitors will remain unchanged. For example, in the second comparison, the largest capacitor in the high array connected to the higher voltage side will be switched from V_ref_ to ground. Additionally, the largest capacitor in the low array connected to the lower voltage side will be switched from ground to V_ref_. The comparator then performs a second comparison and outputs the result of the second comparison (D_2_). This process continues until the (N − 2)th comparison is completed. Throughout the switching process, the common-mode voltage remains constant. Based on the capacitive DAC structure, the energy needed to charge and discharge the capacitor is known as the switching energy, and it plays a crucial role in determining the efficiency of different switching schemes. The capacitor array switching energy of the 2nd to (N − 2)th comparison is as follows:

(1) $$E_{i}=\left[\left(1-2D_{i-1}\right)\sum_{j=1}^{i-1}\left(2D_{j}-1\right)\cdot 2^{N-i-j-2}+2^{N-i-2}\right]{\mathrm{CV}}_{\mathrm{ref}}^{2}$$

(N − 1)th comparison: The first capacitor in the unit array on the higher voltage side switches from V_cm_ to ground and the first capacitor on the lower voltage side switches from V_cm_ to V_ref_, based on the result of the previous comparator. Then, the comparator performs the comparison and outputs the result (D_N−1_). From this stage onward, the DAC utilizes an additional reference voltage V_cm_ to perform more bits of conversion, resulting in area savings and increased efficiency. The capacitor array switching energy in the (N − 1)th comparison is as follows:

(2) $$E_{\left(N-1\right)}=\left[\left(1-2D_{N-2}\right)\sum_{j=1}^{N-2}\left(2D_{j}-1\right)\cdot 2^{-j-1}+\frac{1}{2}\right]{\mathrm{CV}}_{\mathrm{ref}}^{2}.$$

Nth Comparison: In this comparison, the last capacitor in the unit array connected to the higher voltage side is switched from V_cm_ to ground. Meanwhile, the capacitor on the lower voltage side remains unchanged, and the comparator outputs the result of the final comparison (D_N_). The switching energy of the capacitor array in this comparison is as follows:

(3) $$E_{N}=\left[D_{N-1}\cdot \sum_{j=1}^{N-2}\left(1-D_{j}\right)\cdot 2^{-j-1}+\left(1-D_{N-1}\right)\cdot \sum_{j=1}^{N-2}D_{j}\cdot 2^{-j-1}\right]{\mathrm{CV}}_{\mathrm{ref}}^{2}$$

For N-bit resolution, assuming equal probability of each code appearance, the average switching energy of the capacitor array is as follows:

(4) $$E_{\mathrm{average}}=\left[\sum_{i=2}^{N-1}\left(2^{N-i-2}-2^{N-2i-1}\right)+2^{-2}-2^{-N}\right]{\mathrm{CV}}_{\mathrm{ref}}^{2}$$

**Figure 4:**
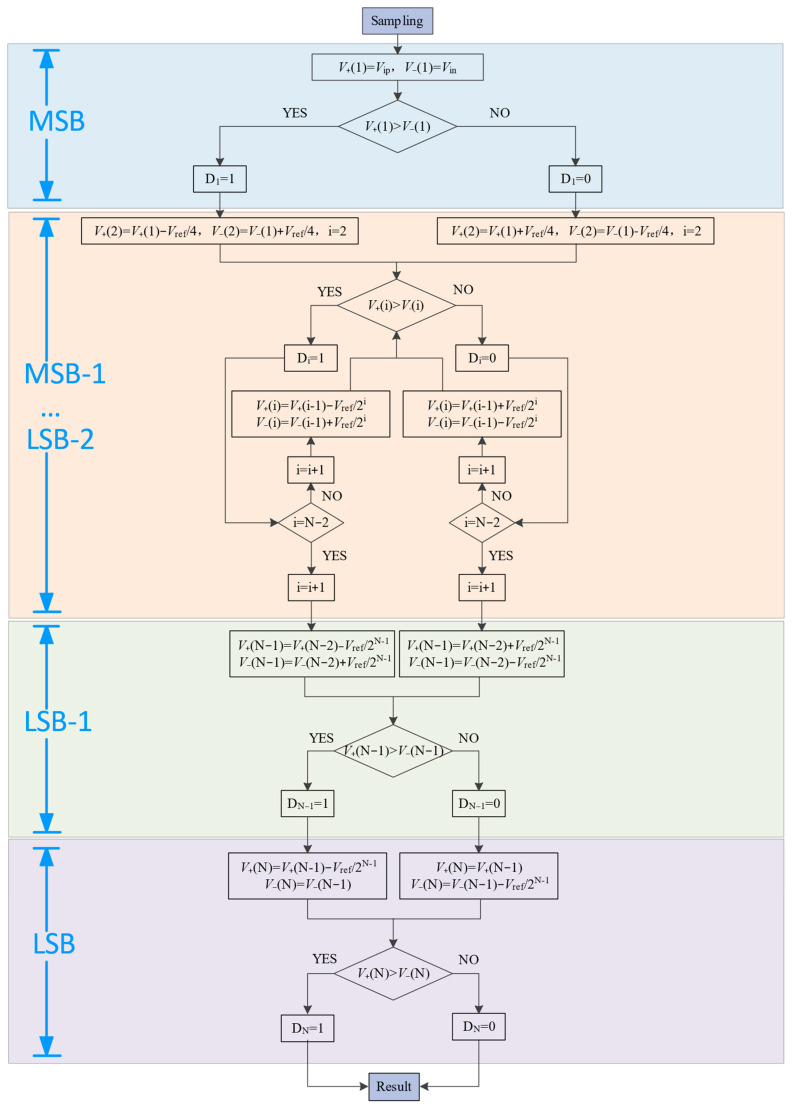
The flow chart of the proposed switching scheme.

Figure 4′s flowchart illustrates the proposed switching scheme. It depicts the comparator’s role in determining the value of the ith bit by generating its corresponding reference voltage. The flowchart also details the DAC switching process, the comparison logic order, and the voltage offset of the capacitor array.

**Figure 5:**
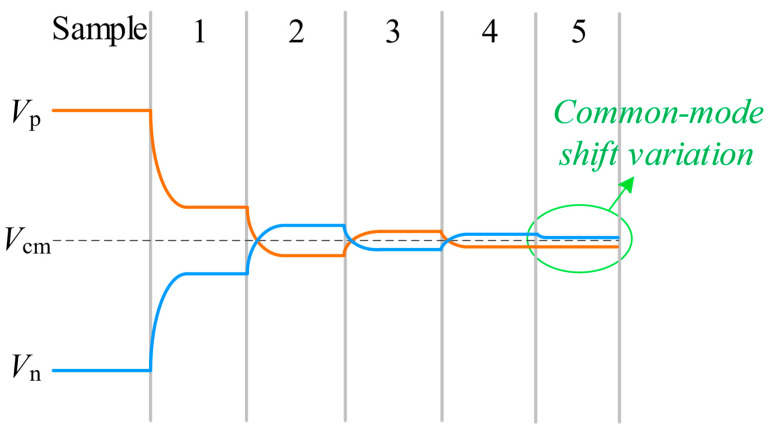
Waveform of the proposed switching scheme.

Figure 5 displays the successive approximation waveforms of the suggested switching scheme. The common-mode voltage remains at V_cm_ from the initial comparison to the (N − 1)th comparison, with only the last comparison shifting the common-mode voltage by one LSB.

**Figure 6:**
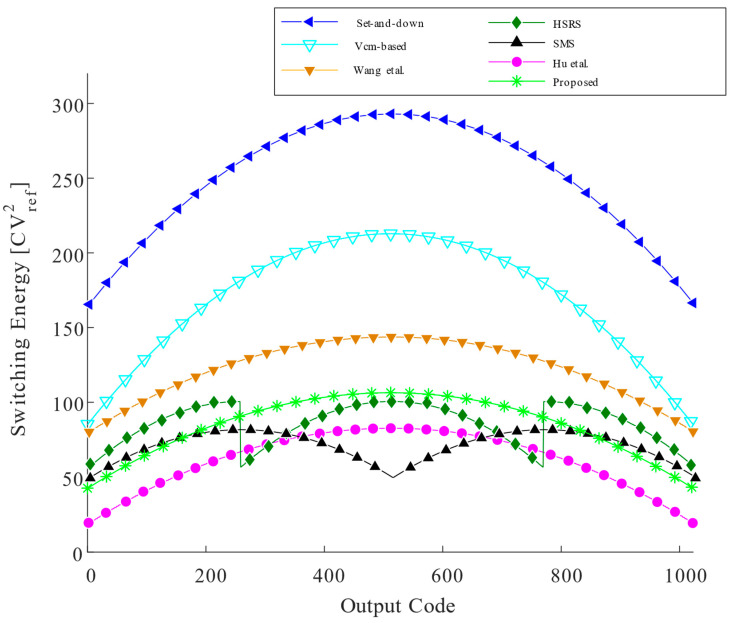
Switching energy against output codes [9,10,11,12,13,14].

Various switching schemes for a 10-bit SAR ADC are simulated. The switching energy at each output code for different switching schemes is illustrated in Figure 6. The proposed scheme has more advantageous average switching energy.

For a 10-bit case, the set and down based on capacitive top-plate sampling and the monotonic capacitor switching procedure reduces the average switching energy to 255.5 $CV_{ref}^{2}$. The set and down [9] is more efficient than the conventional architecture, and the V_cm_-based switching scheme [10] based on the common-mode charge recovery switching method and the use of a third reference voltage, V_cm_, further reduce the switching energy. Wang et al. [11] applied a C-2C dummy capacitor based on the monotonic capacitor switching procedure. The HSRS switching scheme [12] in the first 2 MSBs, which account for a large proportion of energy consumption, achieves zero energy consumption without using any auxiliary circuit. Each of the above two switching schemes achieves 90.61% and 92.2% switching energy reduction compared with the conventional scheme. The shifted monotonic switching scheme (SMS) [13] achieves an average switching energy of 63.75 $CV_{ref}^{2}$ with a reduction of 75% compared to the set and down. Hu et al. [14] proposed a conversion scheme, reducing the switching energy consumption of all comparisons, except for the first 2 MSBs which do not consume switching energy. The average switching energy of the proposed capacitor-splitting low-complexity and energy-efficient switching scheme for a 10-bit SAR ADC is 85.08 $CV_{ref}^{2}$. Compared to the conventional switching scheme [8], the average switching energy is reduced by 93.76%. Based on capacitor-splitting, additional reference voltage V_cm_, and common-mode techniques, the proposed switching scheme does not achieve a lower switching energy than [13,14] but solves the problem of large common-mode voltage shift and high V_cm_ accuracy requirements, and achieves the trade-off between complexity and energy efficiency with low switching energy.

Table 2 presents a comparison of various switching schemes for 10-bit SAR ADCs. These switching schemes [9,11,12,13,14] are highly energy-efficient but suffer from a significant common-mode shift or V_cm_ accuracy, which adversely affects DAC accuracy. In contrast, the proposed DAC switching scheme exhibits a common-mode shift of only one LSB, which is smaller than the common-mode shifts observed in switching schemes [11,12,13,14]. Moreover, the V_cm_ accuracy has a minimal impact on the DAC accuracy.

### 2.2. Bootstrapped Sampling Switch

**Figure 7:**
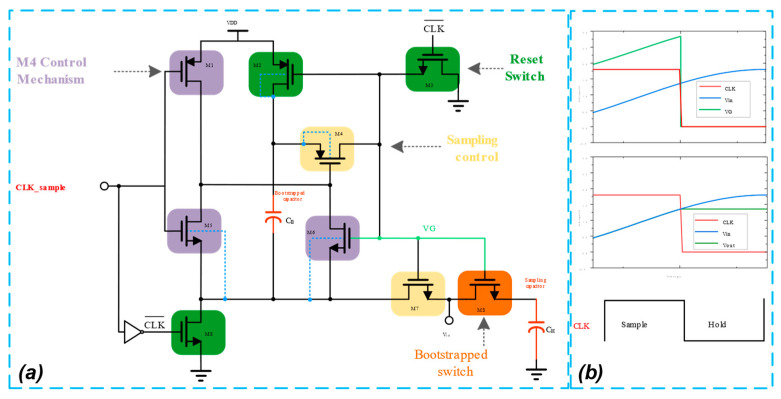
Bootstrapped sampling switch. (**a**) Bootstrapped sampling schematic. (**b**) Transient simulation.

The proposed SAR ADC scheme is a differential structure; in order to improve the performance of the sampling switch and reduce the sampling error, the gate voltage bootstrap switch is used as the sampling switch. The differential sampling structure reduces the common-mode effect of the non-idealities of the bootstrap circuit [26], thereby increasing the accuracy of the sample-and-hold circuit. PMOS or NMOS switches have excessive on-resistance when the input signal is close to VDD or the ground and have poor linearity. In contrast, bootstrap switches possess on-resistance that remains constant regardless of the input signal amplitude, resulting in a higher degree of linearity [27] and a higher spurious-free dynamic range (SFDR) value. The gate voltage bootstrap switch circuit structure is shown in Figure 7a. C_B_ represents the bootstrapped capacitor, C_H_ represents the sampling capacitor, and MS represents the sampling switch tube. The control logic of the sampling switch includes the reset switch, M4 control mechanism, and sampling control. The timing control logic details of the sampling switch are shown in Figure 7b, with the gate voltage bootstrapping process divided into two stages: sampling and holding.

Sampling stage: The sampling signal CLK_sample is high, and the sampling switch is closed; M5 pulls down the gate potential of M4, and the voltage VDD on C_B_ is added to the gate of MS through the M4 tube so that the gate source voltage of MS maintains a constant voltage VDD independent of the input signal, and thus the on-resistance of the MS8 tubes maintains a constant value independent of Vin. Charge conservation is utilized for the gate capacitance of the MS, the CB, and other parasitic capacitances, resulting in the voltage at the VG point being VDD + Vin and the output at the VOUT side being Vin. It should be noted that the back gates of the M2 and M5 need to be connected to the upper board of the C_B_ in order to avoid the latch-up effect.

Holding stage: The sampling signal CLK_sample is low, and the sampling switch is reset; M3 is turned on, and the gate potential of M2 is pulled down to zero scale, at which point the bootstrap switch MS is in the off state. At the same time, M2 and M8 are turned on and capacitor C_B_ is charged to VDD. During the charging of C_B_, M4 and M7 isolate the capacitor from the switch MS, and the node VG is low.

**Figure 8:**
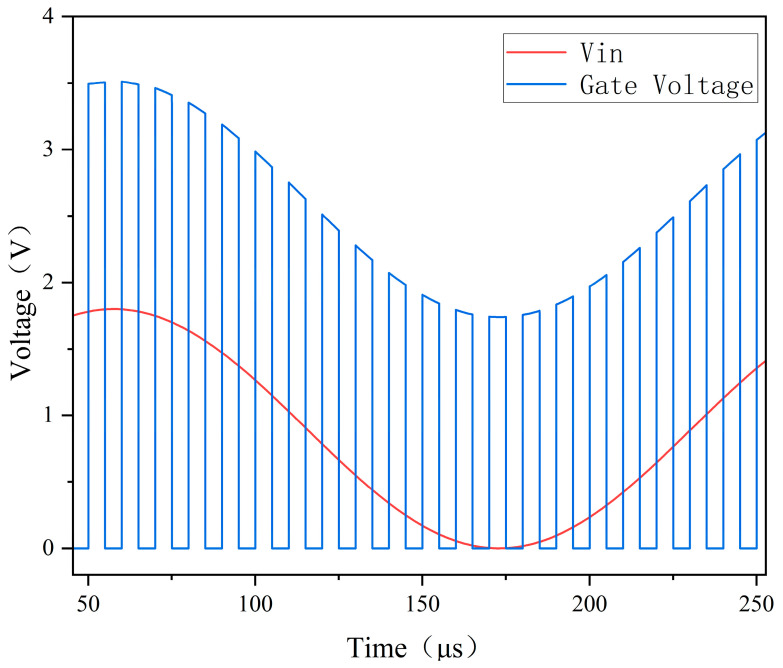
Simulation of bootstrapped sampling switch.

The bootstrap switch is used to create a sample-and-hold circuit, and pre-simulation is performed. As depicted in Figure 8, the output waveform of sinusoidal time-domain transient simulation reveals that when the sampling signal CLK_sample is high, the control signal VG of the sampling switch ascends in a bootstrap manner and fluctuates with the input signal Vin. The waveforms of VG and Vin essentially maintain a constant difference, i.e., the gate voltage of transistor MS remains unchanged with the input signal Vin during sampling. This reduces the sampling error and achieves the gate voltage bootstrap function required by the design.

**Figure 9:**
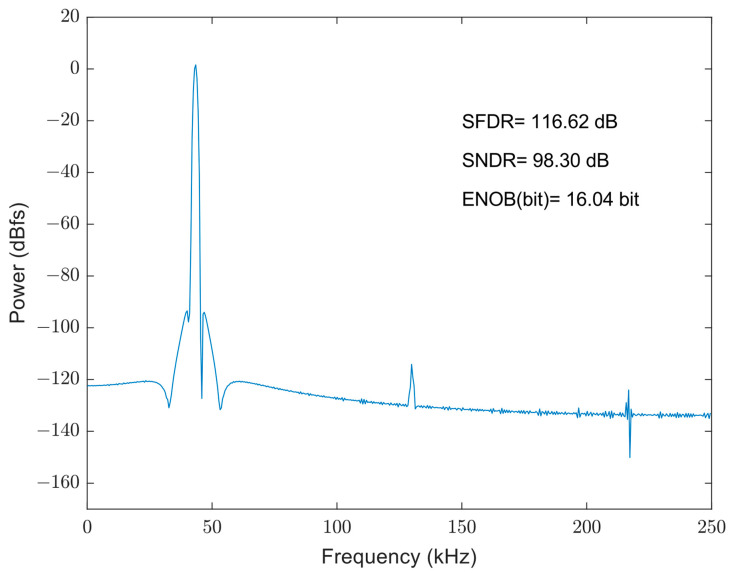
FFT of bootstrapped sampling switch.

Figure 9 illustrates the spectral analysis results of the bootstrapped sampling switch. The FFT plot of the output waveform after the gate voltage bootstrap switch shows good noise suppression, with most of the bottom noise below −130 dB. The signal-to-noise distortion ratio (SNDR) of the sampled output waveform is 98.30 dB, exhibiting good linearity and meeting the requirements of the designed 10-bit ADC. Additionally, the gate voltage bootstrap switch does not consume quiescent operating current, thereby having a small effect on the overall power consumption of the ADC. Consequently, the bootstrap switch design fulfills the necessary criteria.

### 2.3. Dynamic Comparator

**Figure 10:**
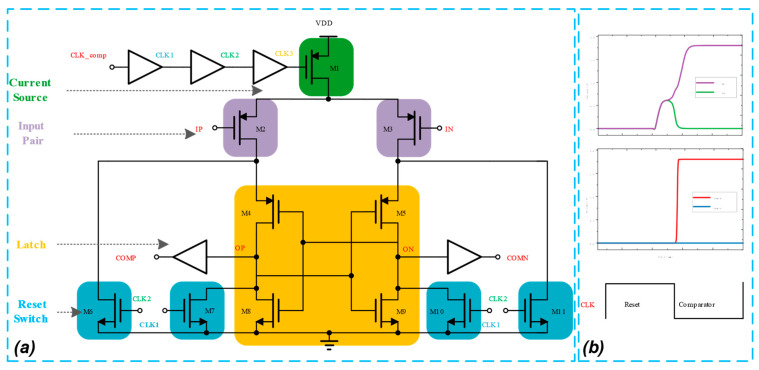
Simulation of dynamic comparator. (**a**) Comparator schematic. (**b**) Transient simulation.

As one of the key modules of the SAR ADC, the performance of the comparator, particularly its speed, power consumption, noise, and misalignment, significantly impacts the overall performance of the SAR ADC circuit and, consequently, the accuracy of the SAR ADC’s digital code output. Dynamic comparators are well suited for low-power, high-speed ADCs [28]. The proposed SAR ADC utilizes a dynamic comparator with self-shutdown control logic, employing a PMOS transistor input pair which offers improved noise immunity at higher operating frequencies [29], as shown in Figure 10a. In order to reduce the noise effect [30] and extend the signal amplification time, the comparator introduces three 50 ps delay buffers. During the comparison’s initiation, only M1′s switching action introduces minimal noise. Due to its lack of static current consumption, a dynamic comparator is suitable for an energy-efficient design. The comparator’s operation is governed by various clock control signals: CLK_comp manages the comparator’s switching state, while CLK1 and CLK2 control the conduction state of the reset control tubes M7, M10, M6, and M11, and CLK3 governs the conduction state of the current source M1. The comparator’s operation is divided into two stages: reset and comparison.

Reset stage: The clock control signals are high, M1 is cut off, the path connecting ON and OP to VDD is disconnected, and the latch output is reset to a low state.

Comparison stage: The falling edge of CLK triggers the comparison process, with VDD charging the ON node through M1, M2, and M4, and the OP node through M1, M3, and M5. The speed of charging depends on the voltage of IP and IN. The input transistors compare input signals and collect charges on nodes OP and ON. If IP is greater than IN, varying currents flow into the cross-coupled inverters M4, M8 and M5, M9. Under the influence of positive feedback, the voltage in the final OP node will increase, while the ON node voltage will decrease, causing COMP to go high and COMN to go low. During comparator operation, if IP is less than IN, the output of the COMP will decrease, while the output of COMN will increase, resulting in a corresponding 0/1 output which completes the comparison process. Additionally, since there is no DC path from VDD to ground, only dynamic power is consumed.

The transient simulation of the comparator is depicted in Figure 10b. The differential input signals IP and IN are 925 mV and 875 mV, respectively. As CLK drops to a low level, the input transistors compare the input signals and accumulate charges on nodes OP and ON. Initially, OP and ON raise their voltages simultaneously. Subsequently, with positive feedback, eventually the OP node voltage increases and the ON node voltage decreases. Finally, the comparator generates the output of the comparison result, where one of COMP and COMN will be high and the other will be low.

### 2.4. Dynamic SAR Logic

The bit conversion of the SAR ADC is under the control of the SAR logic circuit. This circuit uses the comparator’s output to determine the relevant digital bits and generates a control signal to switch the capacitor array to a new state. At the end of the bit-switching process, the logic block outputs all digital codes. Conventional SAR logic mainly consists of ring counters and shift registers, which require at least 2N flip-flops with a large number of MOS tubes, resulting in high power consumption.

**Figure 11:**
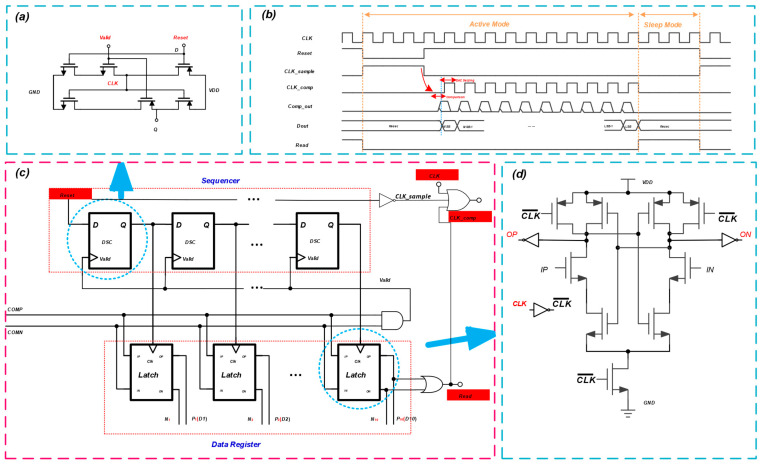
Dynamic SAR logic. (**a**) Dynamic shift controller schematic. (**b**) Timing diagram. (**c**) SAR logic schematic. (**d**) Latch schematic.

The proposed ADC employs dynamic SAR logic to reduce the overall power consumption. As shown in Figure 11c, the switching logic consists of a sequencer and a data register. The sequencer is a shift register that is signalized by a series of dynamic shift controllers (DSCs), as shown in Figure 11a. Once the shift operation is finished, the register’s latch is activated using the set signal reset. The time sequences of the main clock, sampling clock, and digital output of the proposed SAR ADC are shown in Figure 11b. When the last dynamic shift controller in the sequencer turns on, it resets the sequencer and starts a new conversion cycle. The data register consists of dynamic latches that latch the comparator output from high to low. Dynamic latches can latch the differential output of the comparator and output the data differentially, simplifying the logic circuit. The latch schematic details are shown in Figure 11d.

### 2.5. DAC Driver Circuit Design

**Figure 12:**
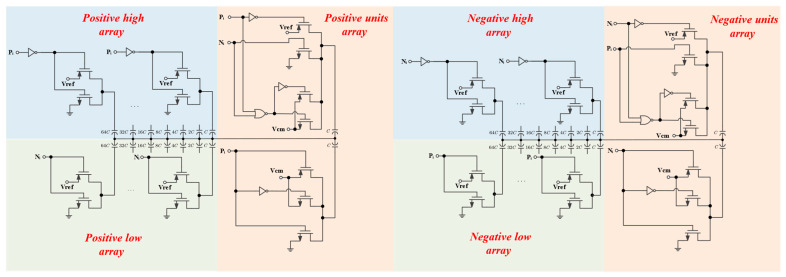
10-bit SAR ADC control logic of the proposed switching scheme.

The control logic circuit is implemented using logic gates and switches. Figure 12 depicts the control logic circuit of the DAC in a 10-bit SAR ADC with the proposed switching scheme. In order to reduce the complexity and power consumption of capacitive drive circuits, the CMOS inverters are mainly used for capacitive driving. Since the last two capacitors use V_cm_ as the reference voltage, the drive needs to add a transmission gate. Figure 12 outlines the drive circuitry and corresponding capacitance for every sub-array in the DAC capacitor array. P_i_ and N_i_ refer to the comparison result of the ith (i = 1–10) bit, where N_i_ is 0 when P_i_ is 1 and vice versa. The 1st bit represents the MSB in a 10-bit SAR ADC. The control logic is simple to implement thanks to the less complex switching scheme. Most of the capacitors are driven by a CMOS inverter structure, and the LSB-1 capacitor is driven by a hybrid structure combining a CMOS transmission gate and a CMOS inverter. Compared with the control logic in Refs. [10,12,13], the complexity of the control logic circuit for the proposed switching scheme is low. With the development of process technology, the power consumption and area of the control logic become small enough.

## 3. Analysis of Results

The SAR ADC was designed and simulated utilizing 180 nm CMOS technology. The size of the unit capacitance C in the capacitor array is set to 4 μm × 4 μm, and C = 17.2 fF. According to the working principle of the logic analyzer, the logic analyzer function module is written in Verilog-A hardware description language for saving the simulation data and then processed.

**Figure 13:**
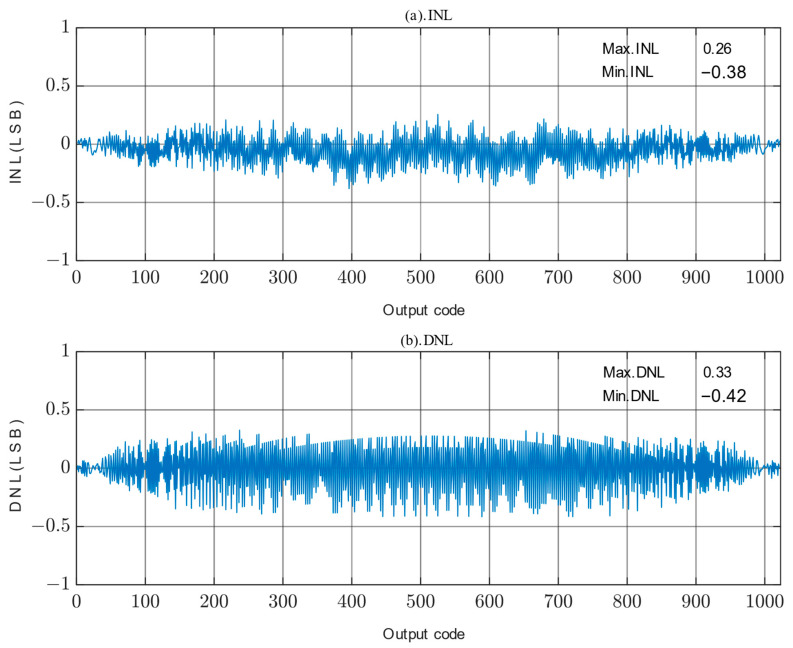
DNL and INL of proposed SAR ADC. (**a**) INL. (**b**) DNL.

The static parameters are tested using the Code Density Test (CDT) method [31]. The simulation parameters are as follows: the power supply is 1 V and the sampling rate is 200 kS/s. The simulated differential non-linearity (DNL) and integral non-linearity (INL) of the proposed SAR ADC are shown in Figure 13; the peak DNL and INL are −0.42 LSB~+0.33 LSB and −0.38 LSB~+0.26 LSB, respectively, which are less than 0.5 LSB, so the designed circuit satisfies the static characterization requirement. The static performance of the proposed SAR ADC is somewhat limited by capacitor mismatch resulting from process gradient errors.

**Figure 14:**
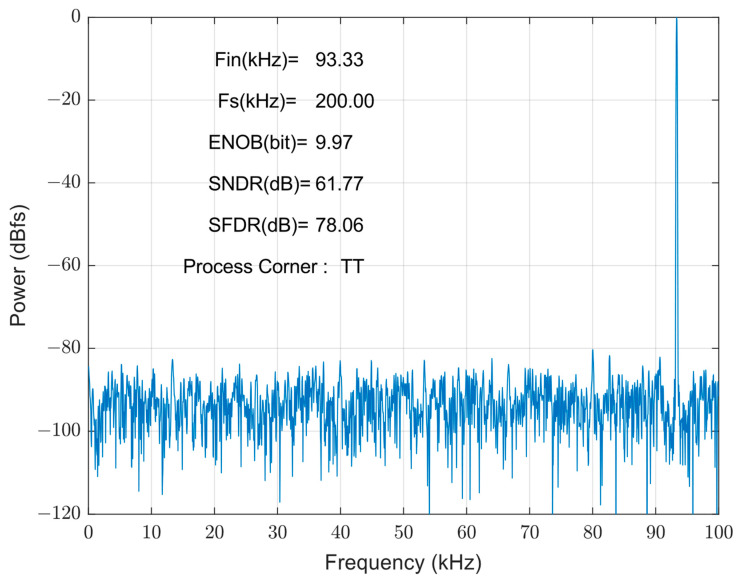
FFT of proposed SAR ADC at the TT process corner.

**Figure 15:**
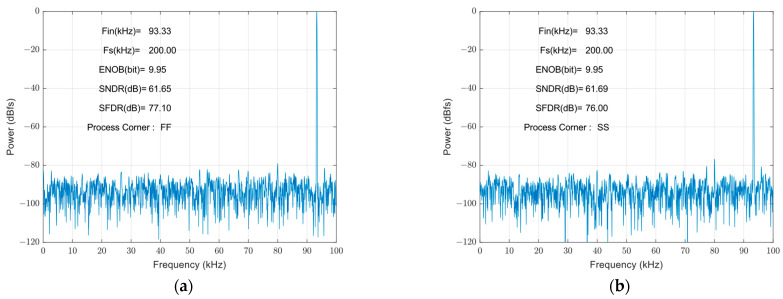
Process corner analysis: (**a**) FF process corner; (**b**) SS process corner.

Figure 14 shows the FFT spectrum of the proposed SAR ADC: the harmonic components are not significant. The ADC achieves a 78.06 dB spurious-free dynamic range (SFDR) and a 61.77 dB signal-to-noise and distortion ratio (SNDR), and the effective number of bits is 9.97 bits. The measured SFDRs demonstrate the good linearity of the proposed bootstrap S/H. The actual effective bits are affected by non-ideal factors, such as noise, misalignment, capacitance mismatch, etc., and the errors caused by these factors are within the acceptable range. Figure 15 shows the FFT of the SAR ADC for different process corners. The ADC achieves a 61.65 dB SNDR and a 77.10 dB SFDR at a process corner of FF, and a 61.69 dB SNDR and a 76.00 dB SFDR in the SS case, with the effective number of bits being 9.95 bits in both cases. Through the simulation and analysis of the process corners, it can be seen that the proposed scheme has a more stable performance under different process conditions, and the circuit has a certain degree of reliability.

**Figure 16:**
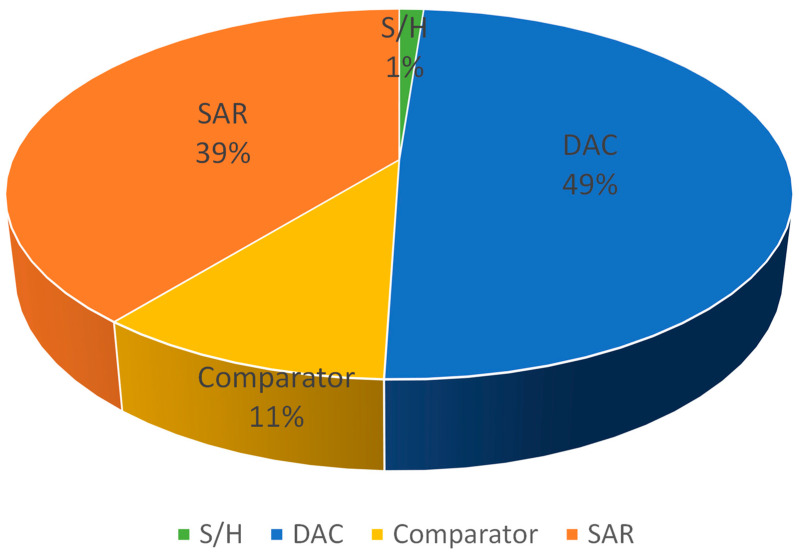
Power breakdown of proposed SAR ADC.

The average total power consumption of the SAR ADC is approximately 4.45 μW. Figure 16′s pie chart displays the power consumption of each component, with the DAC and SAR dissipating the majority of the power. The comparison of the proposed ADC to other advanced SAR ADCs employs the figure of merit (FOM). According to [32], FOM is defined as follows:

(5) $$\mathrm{FOM}=\frac{\mathrm{Power}}{2^{\mathrm{ENOB}}\times F_{s}}$$

where *F_s_* is the sampling frequency, and ENOB is the effective number of bits of the Nyquist input, while Power is the total power consumption of the SAR ADC. The proposed SAR ADC achieves an FOM of 22.2 fJ/conv.-step, which is competitive.

The performance summary of some SAR ADCs [21,22,33,34,35,36] along with the circuit-level simulation results of this work are summarized in Table 3. The suggested ADC is clearly more efficient in terms of power usage and other metrics. It is highly energy-efficient, low in complexity, and small in area. Therefore, this ADC is a more advantageous choice in power and area-constrained wearable biosensor systems. The proposed ADC has many application possibilities since many biosensor devices need to detect analog signals. Moreover, performance can be further improved by using more modern CMOS technology. 

## 4. Conclusions

This paper presents a low-power SAR ADC designed for high-efficiency wearable biosensor applications. The utilization of a capacitor-splitting energy-efficient switching scheme effectively reduces energy consumption. Based on capacitor-splitting, additional reference voltage V_cm_, and common-mode techniques, the proposed switching scheme achieves 93.76% less switching energy compared to the conventional scheme. Furthermore, most of the capacitors have only two reference voltages, accomplishing low complexity, and the accuracy of V_cm_ has a very low impact on the accuracy of the DAC. The proposed DAC switching scheme has a common-mode shift of one LSB, reducing the design complexity of the comparator. In addition, the simulation results demonstrate that the ADC achieves a 61.77 dB SNDR and a 78.06 dB SFDR and consumes 4.45 μW of power in a 180 nm process with a 1 V power supply, a full-swing input signal frequency of 93.33 kHz, and a sampling rate of 200 kS/s.

## Figures and Tables

**Table 2:** Comparison of different switching schemes of a 10-bit SAR ADC.

Switching Scheme	Average Energy ($CV_{ref}^{2}$)	Energy Saving (%)	Sensitivity to the Accuracy of V_cm_	Number of References for Each Capacitor	Maximum Common-Mode Variation
Conventional [8]	1363.3	Reference	No	2	0 LSB
Monotonic [9]	255.5	81.26%	No	2	512 LSB
V_cm_-based [10]	170.2	87.52%	Very high	3	0 LSB
Wang et al. [11]	128	90.61%	No	2	512 LSB
HSRS [12]	106.2	92.20%	Very high	3	256 LSB
SMS [13]	63.75	95.32%	Very high	3	768 LSB
Hu et al. [14]	63.56	95.34%	Very low	2	256 LSB
Proposed	85.08	93.76%	Very low (only LSB and second LSB)	2 (all bits except second LSB)	1 LSB

**Table 3:** Performance comparison of proposed SAR ADC.

Parameter	[21] *	[22] *	[33] *	[34]	[35]	[36] *	This Work *
Process (nm)	180	180	180	180	180	180	180
Resolution (bits)	14	10	10	12	10	10	10
Sampling Rate (MS/s)	0.01	1	1	0.1	0.5	2	0.2
Supply Voltage (V)	1.8	0.5	1.8	1	1	1.8	1
SNDR (dB)	84.50	61.96	58.9	65.3	57.38	59.59	61.77
ENOB (bits)	13.80	7.69	9.5	10.55	9.24	9.65	9.97
DNL (LSB)	0.5	−0.82/0.9	−0.40/0.36	−0.19/0.19	−0.72/0.50	−0.23/0.23	−0.42/0.33
INL (LSB)	0.42	−1.31/1.06	−0.46/0.36	−0.16/0.16	−0.88/0.85	−0.23/0.23	−0.38/0.26
Power Consumption (µW)	19.5	13.99	131	25	14.2	41.92	4.45
FOM (fJ/conv. Step)	140	67.7	181	165	47	26.9	22.2

* Simulated results.

## Data Availability

Data are contained within the article.
